# Sex-Biased Gene Expression and Evolution in the Cerebrum and Syrinx of Chinese Hwamei (*Garrulax canorus*)

**DOI:** 10.3390/genes12040569

**Published:** 2021-04-14

**Authors:** Hua Jiang, Jian-Qing Lin, Li Sun, Yan-Chun Xu, Sheng-Guo Fang

**Affiliations:** 1 MOE Key Laboratory of Biosystems Homeostasis & Protection, State Conservation Centre for Gene Resources of Endangered Wildlife, College of Life Sciences, Zhejiang University, Hangzhou 310058, China; 21807096@zju.edu.cn (H.J.); linjianqing@zju.edu.cn (J.-Q.L.); sunli2012@zju.edu.cn (L.S.); 2 College of Wildlife and Protected Area, Northeast Forestry University, Harbin 150040, China; xu_daniel@163.com; 3 National Forestry and Grassland Administration Research Center of Engineering Technology for Wildlife Conservation, Harbin 150040, China

**Keywords:** de novo assembled transcriptome, chromosome enrichment, Ka/Ks, positive selection

## Abstract

It is common that males and females display sexual dimorphisms, which usually result from sex-biased gene expression. Chinese hwamei (*Garrulax canorus*) is a good model for studying sex-biased gene expression because the song between the sexes is quite different. In this study, we analyze cerebrum and syrinx sex-biased gene expression and evolution using the *de novo* assembled Chinese hwamei transcriptome. In both the cerebrum and syrinx, our study revealed that most female-biased genes were actively expressed in females only, while most male-biased genes were actively expressed in both sexes. In addition, both male- and female-biased genes were enriched on the putative Z chromosome, suggesting the existence of sexually antagonistic genes and the insufficient dosage compensation of the Z-linked genes. We also identified a 9 Mb sex linkage region on the putative 4A chromosome which enriched more than 20% of female-biased genes. Resultantly, male-biased genes in both tissues had significantly higher Ka/Ks and effective number of codons (ENCs) than unbiased genes, and this suggested that male-biased genes which exhibit accelerated divergence may have resulted from positive selection. Taken together, our results initially revealed the reasons for the differences in singing behavior between males and females of Chinese hwamei.

## 1. Introduction

In most species, males and females have approximately identical genomes, but they have many markedly different phenotypic traits, including morphology, behavior, and physiology [1], and most of these sexual differences are denoted as sexual dimorphisms [2]. Sexual dimorphism results from natural selection or sex selection for characteristics that have different fitness optima between the sexes [3]. It is assumed that most sexually dimorphic characteristics result from the difference in gene expression between males and females [4,5]. Genes expressed only in one sex or at a significantly higher level in one sex than in the other are termed as sex-biased genes [3]. A good deal of sex-biased expression genes exist in the different tissues of mammals, birds, nematodes, and insects [6,7,8,9,10].

The distribution traits of sex-biased genes on chromosomes are nonrandom, and the sex chromosomes (Z or X chromosome) usually provide the platform for their enrichment [6,11,12]. Genes which may be favorably selective to one sex but harmful to the other, and can result in different optimal characteristics between the sexes, are referred to as sexually antagonistic genes [13,14]. These genes may be enriched on the sex chromosome. The number of Z (X) chromosomes in males and females is different. In the homogametic sex, two Z (X) chromosomes exist, but only one Z (X) chromosome exists in the heterogametic sex; this results in Z (X)-linked genes having two copies in the homogametic sex, but only one copy in the heterogametic sex. When a recessive gene emerges on the Z (X) chromosome, a heterogametic sex can select it immediately, regardless of the adaptive cost of the homogametic sex being greater than the adaptive benefits of the heterogametic sex and the frequency of the new recessive gene increasing [3,13]. In addition, because genes on the Z (X) chromosome have approximately twice the chance of being selected in a homogametic sex than those on the heterogametic sex, partially or fully dominant genes that are beneficial to the homogametic sex can be accumulated on the Z (X) chromosome [3,13]. Furthermore, the lack of global dosage compensation in birds can explain the uneven distribution characteristics of sex-biased genes on chromosomes [15].

Protein-coding sequences of sex-biased genes, especially male-biased genes, have higher rates of divergence than unbiased genes, and this is common among various species with notable exceptions [3,16]. For example, an early study of the common fruit fly (*Drosophila melanogaster*) revealed that male-biased genes exhibit faster rates of evolution in protein coding sequences [17], but research on gonadal tissues and the carcasses of four species of mosquitoes suggested that female-biased genes have higher rates of evolution [18]. Furthermore, in the gonads of adult chickens (*Gallus gallus*), male-biased genes show higher rates of divergence, but in late embryonic development, the female-biased genes in both gonad and brain tissue exhibit faster evolution rates [19,20]. The high rates of divergence of sex-biased genes can arise from natural selection, sex selection, and relaxed selective constraint [17,21,22].

The vocal behavior of birds, especially songbirds, contains striking sex differences because of sex differences in the cerebrum and syrinx tissue [23,24,25]. With the rapid development of high-throughput RNA sequencing technology (RNA-Seq), an increasing number of transcriptomic studies on non-model organisms have been conducted to study the genes with expression differences between sexually dimorphic traits [26]. Chinese hwamei, also called melodious laughingthrush (*Garrulax canorus*), is a popular caged bird in China because of the attractive song of the male Chinese hwamei [27,28]. In this research, we identified the sex-biased genes in the cerebrum and syrinx tissue of Chinese hwamei by RNA-Seq. In addition, we studied the expression patterns of sex-biased genes and their distribution characteristics in the genome and then compared the rates of divergence of sex-biased and unbiased genes.

## 2. Materials and Methods

### 2.1. Sample Material and RNA-Sequencing

Two male and two female Chinese hwamei cerebrum and syrinx samples, which were collected at about five months old and have been stored in State Conservation Centre for Gene Resources of Endangered Wildlife, were prepared to extract total RNA. This study was given approval by the Institutional Review Board of Experiment Animals Management and Ethics of Northeast Forestry University (NO. 20190903). We used TRIzol (Invitrogen, Carlsbad, CA, USA) to extract the total RNA according to the manufacturer’s protocol. We used the Agilent Bioanalyzer 2100 (Agilent Technologies, Santa Clara, CA, USA) to check RNA integrity number (RIN) and RNA samples with RIN ≥ 6.8 were used for cDNA library construction.

Double-stranded specific cDNA libraries were constructed from each sample using a NEBNext® Ultra™ Directional RNA Library Prep Kit for Illumina® (NEB, Ipswich, MA, USA), following the manufacturer’s protocol. The library concentration was quantified by a Qubit 2.0 Fluorometer (TFS, Waltham, MA, USA). The library quality was checked by the Agilent Bioanalyzer 2100. Sequencing was conducted on a HiSeq 2500 (Illumina, San Diego, CA, USA) in a paired-end 150 bp mode. The mRNA-Seq data generated in this work were deposited in the NCBI SRA database under BioProject accession number PRJNA698077.

### 2.2. De novo Transcriptome Assembly

Poor quality reads, such as reads with adapters and unknown nucleotides were trimmed using fastp (version 0.20.0, Shifu Chen, Shenzhen, Guangdong, China). Reads with a Phred score ≤ 20 bases representing more than 50% of the total read length were also filtered out. After filtering, the *de novo* assembly of high-quality reads from all samples was performed by Trinity (version v2.4.0, Manfred G. Grabherr, MA, USA) with default parameters except a min_kmer_cov of 3 [29,30]. Unigenes were identified using Corset (version 4.6, Nadia M Davidson, Melbourne, VIC, Australia) for further analysis [31]. Reads from each sample were realigned back to the reference transcriptome by bowtie2 (version 2.4.1, Ben Langmead, College Park, MD, USA) [32], and raw read counts of each unigene were obtained using RSEM (RNA-Seq by Expectation-Maximization, version v1.2.15, Bo Li, Madison, WI, USA) [33]. The completeness and accuracy of the assembly results were explored using BUSCO (version 3.1.0, Felipe A. Simão, Geneva, Switzerland) [34].

### 2.3. Transcriptome Annotation

The assembled Chinese hwamei unigenes were annotated against NCBI non-redundant protein sequences (Nr), Eukaryotic Orthologous Groups of proteins (KOG), and Swiss-Prot databases by diamond (version v0.8.22, Benjamin Buchfink, Tübingen, Germany) with an e-value of 1 × 10^−5^ [35]. All unigenes were searched against the NCBI nucleotide (Nt), Protein family (PFAM), Kyoto Encyclopedia of Genes and Genomes (KEGG), and Gene Ontology (GO) databases using BLAST (version v2.2.28+, Stephen F. Altschul, University Park, PA, USA) with an e-value of 1 × 10^−5^ [36], hmmscan (version HMMER 3, Janelia Farm, Ashburn, VA, USA) with an e-value of 0.01 [37], KAAS (version r140224, Yuki Moriya, Uji, Kyoto, Japan) with an e-value of 1 × 10^−10^ [38], and blast2go (version b2g4pipe_v2.5, BioBam Bioinformatics, Valencia, Spain) with an e-value of 1 × 10^−6^ [39], respectively. Likely coding regions of each unigene were predicted using TransDecoder (version 5.5.0, Brian J. Haas, MA, USA) with default parameters.

### 2.4. Differential Expression Analysis

The FPKM (fragments per kilobase of exon per million mapped reads) value was used to quantify gene expression [40]. Genes with FPKM ≥ 1 in at least two biological replicates were termed as actively expressed genes and used for subsequent analysis. We identified sex-biased genes between male and female cerebrum and syrinx samples with a *q* value (adjust *p* value) cut-off of 5% and pAdjustMethod of BH by DESeq2 (version 1.20.0, Michael I Love, Heidelberg, Germany) [41] (Supplementary Document S1). Sex-biased genes with an expression of Fold Change (male/female or female/male) >3 between the sexes and FPKM < 1 in one sex were considered as sex-specific genes.

### 2.5. Chromosomal Enrichment Analysis

We used the assembled Chinese hwamei transcriptome against the latest zebra finch (*Taeniopygia guttata*) genome (assembly accession: GCA_003957565.2) to identify putative chromosome locations of the sex-biased and unbiased genes, as the chromosomes were highly conserved among birds [42,43]. BLAST hits with an e-value > 1 × 10^−10^ were filtered out from the downstream analysis. The observed number of sex-biased genes and the expected number of sex-biased genes in each chromosome were compared. The deviation of the expected number of sex-biased genes was tested by Fisher’s exact test in R software (version 4.0.0, Ross Ihaka, Auckland, New Zealand). A *p*-value ≤ 5% was used to identify the significant depletion or the enrichment of sex-biased genes on a chromosome.

### 2.6. Evolutionary Rates Analysis (Ka/Ks)

One-to-one orthologous sequences between Chinese hwamei and zebra finch were determined by Inparanoid (version 4.0, Kevin P. O’Brien, Tartu, Estonia) using default parameters [44]. A total of 10,938 1:1 orthologous sequences were identified for subsequent analysis. A perl script (Supplementary Document S2) was used to translate amino acid sequences to nucleotide sequences, which were aligned using muscle (version v3.8.31, Robert C. Edgar, Mill Valley, CA, USA) [45]. The nonsynonymous substitution (Ka), synonymous substitution (Ks), and protein substitution rates (Ka/Ks) of the aligned gene pairs were calculated by the KaKs calculator (version 2.0, Zhang Zhang, Seattle, WA, USA) with model MA [46]. To study codon usage bias, we used CodonW (version 1.4.2, John Peden, Nottingham, UK) to calculate the effective number of codons (ENCs) of each unigene. All comparisons between sex-biased and unbiased genes were tested by the Wilcoxon test with R software.

## 3. Results

### 3.1. RNA Sequencing, de novo Assembly of Chinese Hwamei Transcriptome and Transcript Annotation

We sampled eight samples in total (two sexes by two tissues by two replicates). This generated a total of 440,648,252 paired-end 150 bp reads that were generated using the Illumina platform from the eight RNA-Seq libraries of Chinese hwamei. After moving adapter-related and low-quality reads, 427,788,098 (97.08%), clean reads were used for the assembly by the Trinity software [29], resulting in 227,595 transcripts with 450,438,412 bp and 95,962 unigenes with 135,516,981 bp. The mean lengths were 1919 and 1412 bp, and the N50 values were 4437 bp and 3321 bp, respectively (Table 1). More than three quarters of the reads in each sample can be mapped to the transcripts. About 82.9% (single-copy: 81.8%; duplicated: 1.1%) vertebrate orthologs were identified using BUSCO (*n* = 976, C: 82.9% [S: 81.8%, D: 1.1%], F: 12.0% M: 5.1%). A total of 111,894 putative open reading frames (ORFs) were predicted from 40,156 (41.8%) unigenes.

A total of 58,762 (61.23%) unigenes were successfully blasted against at least one of seven databases (GO, Nr, KEGG, PFAM, Nt, Swiss-Prot, and KOG) and 5017 (5.22%) unigenes were successfully blasted against all seven databases. Among the annotated genes, 28,626 (29.82%), 25,265 (26.32%), 10,894 (11.35%), 52,049 (54.23%), 25,265 (26.32%), 20,707 (21.57%), and 8382 (8.73%) were successfully annotated in the Nr, GO, KEGG, Nt, PFAM, Swiss-Prot, and KOG databases, respectively (Table 2).

### 3.2. Sex-Biased Gene Expression Characteristics

For the subsequent analysis of sex-biased gene expression traits, we identified sex-biased genes in Chinese hwamei cerebrum and syrinx using a *q* value of 5%. The number of sex-biased genes in the cerebrum was significantly greater compared with that in the syrinx (Fisher’s exact test, *p* = 1.165 × 10^−5^). Of 19,926 actively expressed genes in the cerebrum, 501 (2.51%) of them exhibited significant differential expression between the sexes, whereas 450 (1.89%) of the 23,753 actively expressed genes in the syrinx were identified as sex-biased genes (Table 3, Supplementary Table S1).

**Figure 1:**
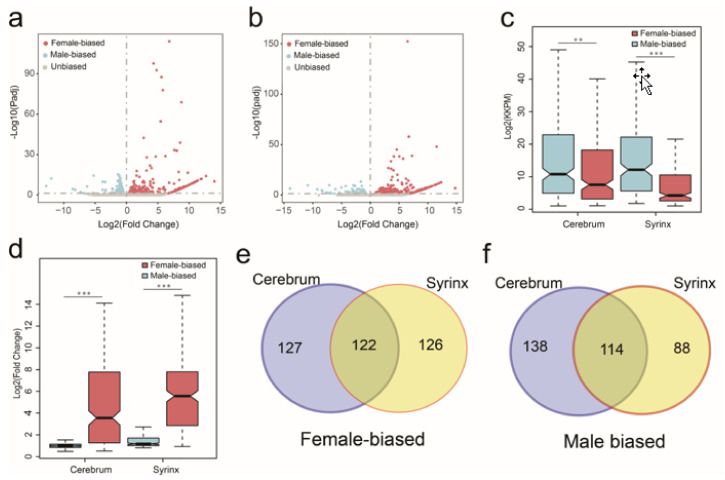
The expression characteristics of sex-biased genes in Chinese hwamei cerebrum and syrinx tissue. The volcano plots show sex-biased gene expression in the cerebrum (**a**) and syrinx (**b**) tissue and the red dots represent the female-biased genes; the light blue dots indicate the male-biased genes, and the gray represents the unbiased genes. Average gene expression level (FPKM) of female-biased genes in female and male-biased genes in male (**c**). Differences in expression magnitude (Fold change) between female-biased and male-biased genes (**d**). Venn diagrams wing the number of female-biased (**e**) and male-biased genes (**f**) in cerebrum and syrinx. Significant difference is represented by *, using Wilcoxon rank sum test, * *p* < 0.05, ** *p* < 0.01, *** *p* < 0.001.

In the cerebrum, the number of female- and male-biased genes showed no significant difference (249 vs. 252, Fisher’s exact test, *p* = 0.9284). In contrast, in the syrinx, the male-biased genes were significantly less than the female-biased genes (202 vs. 248, Fisher’s exact test, *p* = 0.0329) (Table 3; Figure 1a, b). Surprisingly, the number of genes exclusively actively expressed in females (cerebrum: 142 (57.0%); syrinx: 200 (80.6%)) was significantly greater than genes that were actively expressed in males only (cerebrum: 15 (6.0%); syrinx: 32 (15.8%)) in both tissues (Fisher’s exact test, *p* < 2.2 × 10^−16^, both) (Table 3). In addition, in both the cerebrum and syrinx, the expression level of female-biased genes in females was significantly lower than that of male-biased genes expressed in males (Wilcoxon rank sum test, *p* = 0.0014; *p* = 7.65 × 10^−16^, respectively) (Figure 1c), but the magnitude of sex-bias was significantly greater for female-biased genes relative to male-biased genes (Wilcoxon rank sum test, *p* = 5.09 × 10^−10^; *p* < 2.2 × 10^−16^, respectively) (Figure 1d). Our results indicated most female-biased genes were exclusively actively expressed in females, but most male-biased genes were actively expressed in both sexes. Finally, our results suggested that about half the sex-biased genes have the same sex-biased pattern in the two tissues (Figure 1e,f).

### 3.3. Genome Distribution of Sex-Biased Genes

**Figure 2:**
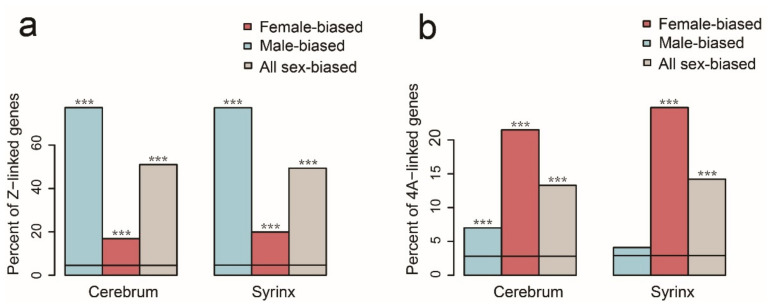
Chromosome enrichment of sex-biased genes: Z chromosome enrichment of sex-biased genes (**a**); and 4A chromosome enrichment of sex-biased genes (**b**). The predicted percentage of sex-biased genes on chromosome Z or 4A (horizontal black solid lines). The actual percentage of sex-biased genes on chromosome Z or 4A (vertical bars). Significant difference is represented by *, using Fisher’s exact test, * *p* < 0.05, ** *p* < 0.01, *** *p* < 0.001.

Chromosomes are quite stable among birds [42,43]; therefore, we used the zebra finch genome to speculate on the genome location of sex-biased genes in Chinese hwamei. In both the cerebrum and syrinx, the sex-biased genes were significantly accumulated on the Z chromosome and 4A chromosome. In the cerebrum, 177 of the 229 male-biased genes were on the Z chromosome (77.3%. Fisher’s exact test, *p* < 2.2 × 10^−16^) and 30 of the 177 female-biased genes were Z-linked genes (16.9%, Fisher’s exact test, *p* = 5.392 × 10^−10^) (Figure 2a). Interestingly, 16 of 229 male-biased genes were 4A-linked genes (7.0%, Fisher’s exact test, *p* = 0.0007) and 38 of 177 female-biased genes were on the 4A chromosome (21.5%, Fisher’s exact test, *p* < 2.2 × 10^−16^) (Figure 2b). In the syrinx, we observed a similar pattern: 132 of 171 male-biased genes were Z-linked genes (77.2%, Fisher’s exact test, *p* < 2.2 × 10^−16^) and 32 of 161 female-biased genes were on the Z chromosome (19.9%, Fisher’s exact test, *p* = 1.597 × 10^−11^) (Figure 2a). Furthermore, 40 of 161 female-biased genes were on the 4A chromosome (24.8%, Fisher’s exact test, *p* < 2.2 × 10^−16^) but male-biased genes were not enriched on the 4A chromosome (4.1%, Fisher’s exact test, *p* = 0.3494) (Figure 2b). Taken together, our results revealed that there are more male-biased genes relative to female-biased genes on the Z chromosome, but surprisingly, on the 4A chromosome, we could observe more female-biased relative to male-biased genes.

**Figure 3:**
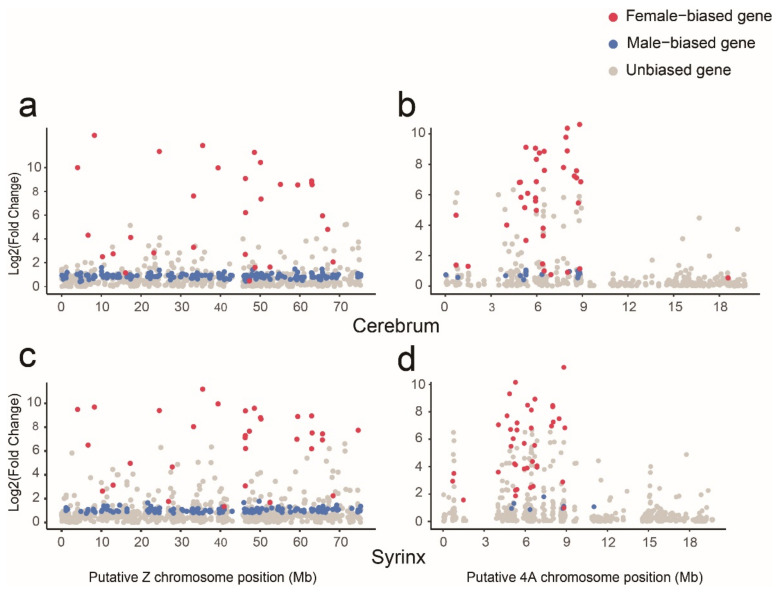
Distribution and expression characteristics of sex-biased genes on chromosomes Z and 4A. The distribution and expression characteristics of the cerebrum (Z (**a**); 4A (**b**)) and syrinx (Z (**c**); 4A (**d**)) sex-biased genes on chromosomes Z and 4A. The vertical axis represents the difference in gene expression magnitude (Fold Change) between males and females. The horizontal coordinates represent the position of the gene on the putative chromosomes Z and 4A.

We further investigated the distribution and the expression characteristics of sex-biased genes on chromosomes Z and 4A. In both cerebrum and syrinx, we observed that the sex-biased genes were almost evenly distributed on chromosome Z (Figure 3a,c), while on chromosome 4A, sex-biased genes were almost all located between 0 and 9 Mb, especially between 3 and 9 Mb (Figure 3b,d). In addition, in both tissues, the differences in the magnitude of gene expression between the sexes on chromosomes Z and 4A were obviously lower for male-biased genes relative to female-biased genes (Figure 3).

### 3.4. Accelerated Protein Evolution and Codon Usage Bias of Sex-Biased Genes

We attained 10,938 1:1 orthologs between Chinese hwamei and zebra finch using Inparanoid 4.0. Among these orthologs, 8882 and 9423 are actively expressed in the brain and syrinx, respectively. To understand the difference in the protein evolution rate, the rates of nonsynonymous (Ka) to synonymous (Ks) replacements (Ka/Ks) were compared. 

**Figure 4:**
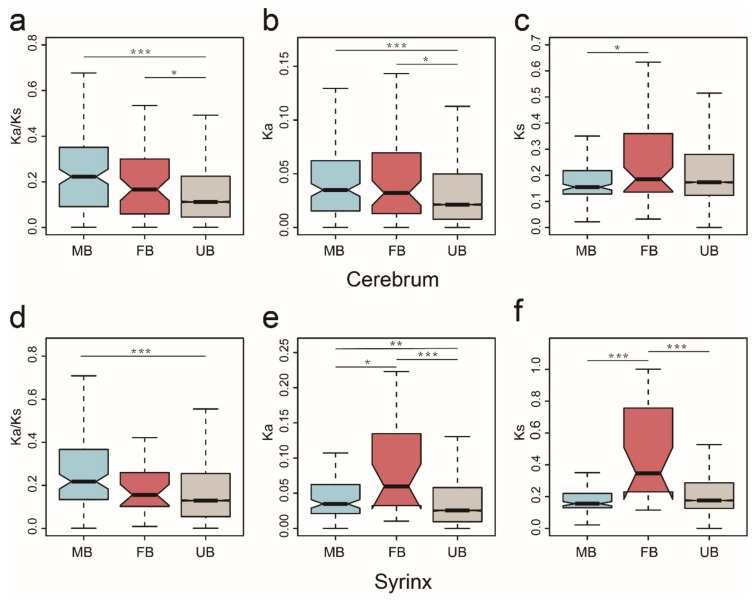
The rates of divergence of sex-biased genes and unbiased genes in hwamei cerebrum and syrinx. Boxplots show the Ka/Ks (cerebrum (**a**); syrinx (**d**)), Ka (cerebrum (**b**); syrinx (**e**)) and Ks (cerebrum (**c**); syrinx (**f**)) distribution trait for the male-biased genes (MB), female-biased genes (FB) and unbiased genes (UB) in the cerebrum and syrinx tissues. Significant difference is represented by *, using Wilcoxon rank sum test, * *p* < 0.05, ** *p* < 0.01, *** *p* < 0.001.

In both the cerebrum and syrinx, we compared the Ka/Ks of sex-biased and unbiased genes and found significantly higher Ka/Ks values in sex-biased genes. In both tissues, male-biased genes had significantly greater Ka/Ks values compared with unbiased genes (Wilcoxon rank sum test, *p* = 8.08 × 10^−12^, *p* = 7.92 × 10^−7^, respectively). However, for female-biased genes, we only observed a significant increase in Ka/Ks values in the cerebrum (Wilcoxon rank sum test, *p* = 0.0410) (Figure 4a,d). Additionally, in both tissues, when compared with unbiased genes, we also observed significantly greater Ka values (Wilcoxon rank sum test, *p* = 1.424 × 10^−5^, *p* = 0.0044, respectively) (Figure 4b,e) and obviously lower Ks values but with no significant difference (Wilcoxon rank sum test, *p* = 0.0815, *p* = 0.0924, respectively) in male-biased genes (Figure 4c,f). In addition, female-biased genes in both tissues also had significantly greater Ka values relative to the unbiased genes (Wilcoxon rank sum test, *p* = 0.0437, *p* = 0.0002, respectively) (Figure 4b,e). Surprisingly, in the syrinx, there were significantly higher Ks values in female-biased genes compared with unbiased genes (Wilcoxon rank sum test, *p* = 4.775 × 10^−5^) (Figure 4f). In summary, our results demonstrated that male-biased genes in both the cerebrum and syrinx have higher evolutionary rates, mainly as a result of significantly elevated rates of Ka, although we could also observe obvious but not significant differences in the decreasing rates of Ks.

**Figure 5:**
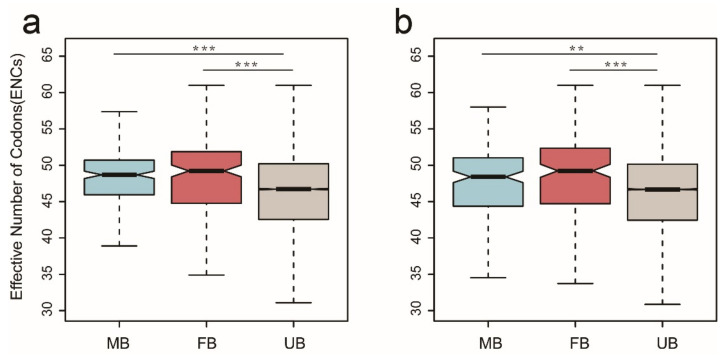
Codon bias of sex-biased and unbiased genes in the cerebrum (**a**) and syrinx (**b**). Male-biased genes (MB); female-biased genes (FB); and unbiased genes (UB). Significant difference is represented by *, using Wilcoxon rank sum test, * *p* < 0.05, ** *p* < 0.01, *** *p* < 0.001.

Codon usage bias occurs in most organisms, and it results from weak nature selection pressure [47,48]. Some studies have demonstrated that sex-biased genes, which have higher rates of evolutionary divergence, have lower codon usage bias than unbiased genes [49,50,51]. Therefore, we used codon usage bias, which estimates the ENCs to validate the above Ka/Ks results. As expected, our results suggest that sex-biased genes have lower codon bias compared with the unbiased genes. In both the cerebrum and syrinx, we observed significantly higher ENCs of male-biased genes relative to unbiased genes (Wilcoxon rank sum test, *p* = 7.754 × 10^−7^, *p* = 0.0025, respectively) (Figure 5a,b). In addition, we also noticed female-biased genes in both tissues showing significantly lower codon usage bias relative to unbiased genes (Wilcoxon rank sum test, *p* = 4.592 × 10^−7^, *p* = 3.251 × 10^−7^, respectively) (Figure 5a,b).

## 4. Discussion

In this study, in order to study the sex-biased gene expression and evolution of the cerebrum and syrinx, eight samples from Chinese hwamei cerebrum and syrinx were used to obtain the *de novo* assembly of Chinese hwamei transcriptome. The N50 and unigene length of transcript can be used to assess the quality of some birds’ *de novo* assembly. Additionally, in our results, the transcript N50 (4437 bp) and average lengths (1919 bp) were similar to or longer than those of other birds, such as the song sparrow (*Melospiza melodia*) [52], little greenbul (*Andropadus virens*) [53] and tree swallow (*Tachycineta bicolor*) [54]. The completeness and accuracy of the assembly results were also similar to tree swallow [54] and blue-winged teal (*Spatula discors*) [55]. This suggests that the assembly quality is great and the assembly transcriptome is enough for downstream analysis.

Many sex-biased expressed genes exist in different tissues of many species [3,16,21]. Although most studies have focused on the gonads [19,22,56], some studies have also found that extensive sex-biased expression exists between the sexes in somatic tissues [57,58,59]. In our research, 2.51% of actively expressed genes in the cerebrum showed sex-biased expression, and a similar proportion has been found in the blue tit (*Cyanistes caeruleus*) [8], common whitethroat (*Sylvia communis)* [60] and zebra finch brain [11]. Our results indicateed that the number of sex-biased genes in the cerebrum is significantly greater than that in the syrinx, suggesting that the cerebrum may exhibit more sexually dimorphic traits.

Interestingly, our results found that in both the cerebrum and syrinx, most female-biased genes were actively expressed exclusively in females, while most male-biased genes were actively expressed in both males and females, although the number of female- and male-biased genes in the cerebrum showed no significant difference. A similar phenomenon has been found in the blue tit brain and the study hypothesized that most female-specific genes are ncRNA genes, while most male-biased genes are coding genes [8]. In addition, our results showed that in both somatic tissues, the magnitude of sex-bias was significantly greater in female-biased genes compared with male-biased genes and shows “feminization”, but the average expression level of female-biased genes in females was significantly lower than that in male-biased genes in males and showed “masculinization”. This differs from zebrafish (*Danio rerio*) and wild turkey (*Meleagris gallopavo*), and both the expression level and magnitude of sex-bias showed “masculinization” [10,61]. This suggests that different organisms show different sex-biased expression patterns.

Sex-biased genes are unevenly distributed on the genome among various clades, especially showing enrichment on the X or Z chromosome in the homogametic sex via a pervasive phenomenon [11,12,62,63,64,65]. Our results exhibited that both female- and male-biased genes in both tissues were significantly enriched on the Z chromosome. Our results followed previous reports, which revealed that not only male-biased genes [8,11,60,62], but also female-biased genes [11] in birds were enriched on the Z chromosome, although female-biased gene enrichment was reported only in the zebra finch brain. Recessive mutations beneficial to the heterogametic sex can be exposed to natural selection and quickly selected [3,13,66]. This might explain why we can observe the accumulation of female-biased genes on the Z chromosome in both Chinese hwamei cerebrum and syrinx. Male-biased genes accumulated on the Z chromosome in both tissues may be driven by two reasons. On the one hand, as partially or completely Z (X)-linked mutations have twice the chance of selection in homogametic sex than in heterogametic sex, homogametic beneficial sex-linked mutations may easily go to fixation [3,13,66]. On the other hand, the insufficient dosage compensation mechanism of the sex-linked genes is a common pattern across many species, including birds [67,68,69,70]. In addition, our results also revealed that female-biased genes are accumulated on the 4A chromosome and were almost all located between 0 and 9 Mb in both the cerebrum and syrinx. It has been demonstrated that the 4A chromosome is a neo-sex chromosome in some Sylvioidea songbirds, and approximately half of it (0–9.6 Mb) is fused with ancestral Z and W chromosomes [71,72,73]. Our results revealed that like other Sylvioidea songbirds, Chinese hwamei may have a sex linkage region between 0 and 9 Mb of the 4A chromosome.

Similarly to the uneven distribution characteristics of sex-biased genes on chromosomes, when compared with unbiased genes, the elevated evolutionary rates of sex-biased genes are observed across many organisms including animals [17], plants [51], and fungi [74]. Here, we observed that male-biased genes in both the cerebrum and syrinx exhibited accelerated divergence, while female-biased genes exhibited only rapid evolution in the cerebrum. Similar results were also found in chicken, both female- and male-biased genes have higher rates of divergence relative to unbiased genes [19,20].

Elevated evolutionary rates of protein sequences in birds are driven by not only positive selection but also relaxed selective constraints [22,26,75]. In our results, both female- and male-biased genes in both the cerebrum and syrinx exhibited significantly lower codon usage bias. These results were consistent with those of the salmon lice (*Lepeophtheirus salmonis*) [50] and the common fruit fly [49]. This indicated the accelerated divergence of sex-biased genes in Chinese hwamei somatic tissues results from positive selection. Additionally, our results showed that more than three quarters of male-biased genes in both tissues are Z-linked genes, and fast-evolving sex-linked genes are associated with the “Fast-Z effect” in birds [76,77]. This may also explain why male-biased genes show rapid evolution.

## Figures and Tables

**Table 1:** Summary of de novo sequence assembly for Chinese hwamei.

	Unigene	Transcript
Total Number	95,962	227,595
Total Length	135,516,981	450,438,412
Mean Length	1412	1979
N50 Length	3221	4437

**Table 2:** Summary of the annotation results.

Databases	Nr	Nt	GO	KEGG	PFAM	Swiss-Prot	KOG	At least one	All
Annotated Unigenes	28,626	52,049	25,265	10,894	25,265	20,707	8382	58,762	5017

**Table 3:** Sex-biased gene expression in cerebrum and syrinx tissues.

	Criteria	Cerebrum	Syrinx	Overlap
Actively Expressed Genes	FPKM ≥ 1 at least two replicates	19,926	23,753	15,360
Male-Biased Genes	*q* < 0.05 and male-biased	252	202	114
Female-Biased Genes	*q* < 0.05 and female-biased	249	248	122
Male-Specific Genes	Male/female > 3 and FPKM < 1 in females	15	32	7
Female-Specific Genes	Female/male > 3 and FPKM < 1 in males	142	200	90

## Data Availability

The zebra finch genome is available from GenBank (assembly accession: GCA_003957565.2). The mRNA-Seq data generated in this work have been deposited in the NCBI SRA database under BioProject accession number PRJNA698077.

## Supplementary Materials

The following are available online at https://www.mdpi.com/article/10.3390/genes12040569/s1, Supplementary Document S1: An R script for obtaining differentially expressed genes (sex-biased genes); Supplementary Document S2: A perl script for translating amino acid sequences to nucleotide sequences; Supplementary Table S1: Raw expressed data and Expression data of sex-biased genes in the cerebrum and syrinx.
