# Supplementary material for: Sex-Biased Gene Expression and Evolution in the Cerebrum and Syrinx of Chinese Hwamei (Garrulax canorus)
Source: Genes (Basel). 2021 Apr 14;12(4):569. doi: 10.3390/genes12040569 (PMC8070764; doi:10.3390/genes12040569)
Supplement: Supplementary file 1 [file genes-12-00569-s001.zip › Supplementary Files/Supplementary Document S1.docx]

Supplementary DocumentS1: An R script for obtaining differentially expressed genes (sex-biased genes). The R version is 3.50 and the DESeq2 version is 1.20.0.

library(DESeq2)

library(readxl)

setwd("E:/research")

Cerebrum<-read_xlsx("all.xlsx",sheet=1)

Cerebrum

Cerebrum <- data.frame(Cerebrum)

rownames(Cerebrum) <- Cerebrum[,1]

Cerebrum <- Cerebrum[,-1]

head(Cerebrum)

Cerebrum<- as.matrix(Cerebrum)

condition <- factor(c(rep("control",2),rep("treat",2)),

levels = c("control","treat"))

condition

colData <- data.frame(row.names=colnames(Cerebrum), condition)

colData

dds <- DESeqDataSetFromMatrix(countData = round(Cerebrum), colData = colData,

design= ~ condition)

dds <- dds[ rowSums(counts(dds))>1, ]

dds <- DESeq(dds)

res <- results(dds, contrast=c("condition", "control", "treat"),

pAdjustMethod = "BH", alpha = 0.05)

summary(res)

diff_gene_deseq2 <-subset(res, padj < 0.05)

diff_gene_deseq2

write.csv(res,file="All_results.csv")
