# Supplementary material for: Sex-Biased Gene Expression and Evolution in the Cerebrum and Syrinx of Chinese Hwamei (Garrulax canorus)
Source: Genes (Basel). 2021 Apr 14;12(4):569. doi: 10.3390/genes12040569 (PMC8070764; doi:10.3390/genes12040569)
Supplement: Supplementary file 1 [file genes-12-00569-s001.zip › Supplementary Files/Supplementary Document S2.docx]

Supplementary Document S2: a perl script for translating amino acid sequences to nucleotide sequences.

#!/usr/bin/perl -w

use strict;

use File::Basename;

die "Usage: perl $0 <pepdir> <cdsdir> <outdir>\n" if (@ARGV<3);

my $dir_pep=shift;

my $dir_cds=shift;

my $outdir=shift;

mkdir $outdir if (! -d $outdir);

my (%pep,%cds,%family,%cdsprank);

opendir DIR, "$dir_pep" || die "Can't open such dir:$!";

my @pep_prank=grep{$_ !~/^\.|\.\.$/} readdir(DIR);

my @cds=`ls $dir_cds`;

foreach my $pepprank (@pep_prank){

next if ($pepprank=~/^(\.)$/);

next unless ($pepprank=~/(2\.fas)$/);

&readfasta ("$dir_pep/$pepprank",\%pep);

&faid_geneid ("$dir_pep/$pepprank",\%family)

}

foreach my $cds (@cds){

&readfasta ("$dir_cds/$cds",\%cds);

}

foreach my $p2 (keys %pep){

my $length=length($pep{$p2});

my $j=0;

for (my $i=0;$i<$length;$i++){

my $is=substr($pep{$p2},$i,1);

if ($is eq "-"){

$cdsprank{$p2} .= "---";

}else {

$cdsprank{$p2} .=substr($cds{$p2},$j,3);

$j +=3;

}

}

}

sub readfasta {

my ($file,$hpep)=@_;

open IN,"$file" || die "Can't open such file:$!";

$/=">";

<IN>;

$/="\n";

while (<IN>){

my $name=$1 if (/(\S+)/);

$/=">";

my $seq=<IN>;

$/="\n";

$seq=~s/\s+//g;

$seq=~s/>$//;

$$hpep{$name}=$seq;

}

close IN;

}

sub faid_geneid {

my ($file,$faid)=@_;

my $nei=basename($file);

my $fd=(split /\./,$nei)[0];

open IN2,"$file"|| die "Can't open such file:$!";

while(<IN2>){

next unless (/>/);

chomp;

$_=~s/>//;

$$faid{$fd} .="$_\t";

}

}

foreach (keys %family){

open OUT,">$outdir/$_.fasta.2.fas";

$family{$_}=~s/\t$//;

my @a=split /\t/,$family{$_};

foreach my $a(@a){

print OUT ">$a\n$cdsprank{$a}\n" if (exists $cdsprank{$a});

}

}
